# Cases Illustrating the Use of the Balsam of Copaiba in Chronic Inflammation of the Mucous Membrane of the Lungs; with Remarks

**Published:** 1827-06

**Authors:** R. La Roche


### *Cases illustrating the Use of the Balsam of Copaiba in Chronic Inflammation of the Mucous Membrane of the Lungs; with Remarks.* By R. La Roche, m.d.

Case I.—Christina P., aged about eighteen years, was attacked, towards the end of September, 1821, whilst in the country, with the characteristic symptoms of inflammation of the lining membrane of the lungs. She was bled, vomited, and purged, by the physician of the place, and kept on a strict antiphlogistic regimen. About two weeks after her first attack she was removed to the city, and placed under my direction. At the first visit, she presented the following discouraging symptoms: violent cough, which was particularly troublesome during the night, and accompanied with a copious expectoration of matter of a dark yellowish-green colour and unpleasant smell, and sinking in water. She was moreover labouring under fever, characterised by a pulse beating 120 strokes in the minute; hot skin, especially of the palms of the hands; hectic flushes, night sweats, &c. &c. She was ordered the usual remedies, with demulcent drinks, mild anodynes at night, and a blister to the chest.

After continuing a few days under this treatment, she was suddenly attacked with hemoptysis, and her fever acquired intensity. For these symptoms she was bled to the amount of ten ounces; ordered fifteen drops of Tincture of Digitalis three times a-day; and the blister was kept in a state of suppuration.

From these means she experienced some relief; the spitting of blood was arrested, and the condition of her pulse and skin improved. Finding, however, that her cough and expectoration continued as before, together with her nocturnal febrile excitement, and observing, moreover, that there existed no sign of irritation of the stomach, she was ordered to take a table-spoonful of Balsam Copaiba mixture three times a-day. Under the use of this medicine and of the Digitalis, the nocturnal febrile symptoms amended, the expectoration improved, and the appetite gradually returned. She was then put under the use of a small quantity of milk for diet, and the mixture was continued.

By persevering in the use of these means, she derived such manifest benefit, that, after a few weeks, she was in a fit condition to be allowed a more substantial diet, and to be permitted to visit her parents in the country; whence, after a few months, she returned in apparent good health, and free from cough, expectoration, or fever. She now enjoys good health, is married, and has borne children.

*Remarks.*—This case, the first of this disease in which I had employed the copaiba, was well calculated to make a strong impression on my mind, and to induce me to have recourse to it on the first favourable occasion that should present itself to my observation. I accordingly soon did so in a case of confirmed phthisis, for which I was consulted, not with a view of curing the patient, (for the disease had already progressed too far to allow me to anticipate a recovery,) but of relieving her troublesome cough and copious expectoration. Nor was I disappointed in my expectation: the cough was in some measure relieved, but in the course of a few months the patient sunk to the grave. The same effects have subsequently been derived in many similar cases, from the same remedy; but yet I am far from wishing to inculcate the idea that, in most cases of confirmed phthisis of either kind admitted by nosologists, the copaiba will be found an effectual and useful remedy, or even a palliative; for I am too well aware that this disease is, in very many cases, accompanied with symptoms indicating irritation, and even inflammation, of the stomach and bowels, and with general fever, which, as we have seen, contra-indicate the use of the remedy.

Case II.—Mrs. P., an old lady, about sixty-five years of age, and who has been subject for many years to frequent attacks of bronchitis, supervening on a chronic state of the same disease, was taken, in November 1823, with high febrile excitement, violent cough, dull pain about the chest, a little oppression, headache, thirst, foul tongue, nausea, &c. The state of her pulse seeming to forbid the use of the lancet, she was puked with Ipecacuanha, her bowels were opened with Senna and Manna; she was kept on low diet, and under the influence of small and repeated doses of Antimony. After a few days her fever abated, but the cough continued very troublesome, and expectoration very copious, yellow, thick, and opaque.

Small nauseating doses of Ipecacuanha, opiates at night, and Tartar-emetic Ointment to the chest, which were now resorted to, seemed to produce a beneficial effect, and were persevered in for some time. Finding, however, that her cough and expectoration were still troublesome, and as her tongue was now perfectly clean, moist, and rather pale, and her digestive organs in a natural condition, she was ordered twenty-five drops of Balsam Copaiba, in a wine-glassful of cold chamomile tea, three times a-day. She continued under the use of this remedy during four weeks, increasing the dose gradually to forty drops, and adding occasionally a few drops of laudanum, to prevent catharsis. She was at the same time recommended a strict milk diet, and, at the end of the period before mentioned, I had the satisfaction of finding that all her unpleasant symptoms had disappeared.

She continued to enjoy perfect health for several months after, since which time I have lost sight of her. When I last saw her, she unhesitatingly informed me that she was better than she had been for a long time before, and attributed her cure to the balsam.

*Remarks.*—I am perfectly aware that by many I shall be accused of attributing, perhaps, too great an agency, in the happy termination of this case, to the copaiba; and that the external revulsive application, together with the other remedies and the abstinence, might have been sufficient to effect the cure, without the aid of this medicine. Admitting, however, that this might have occurred, would we not be warranted, by a parity of reasoning, in calling in question the real efficacy of every article of the materia medica; since few are used alone, and without the assistance of others. But it may be remarked, that the subject of the present observation had been affected before with similar symptoms, and that, although treated by means of the same remedies to which I had recourse, (the copaiba excepted,) she had retained her cough for a number of months, or even years. When this remedy was commenced, the cough was very troublesome, and expectoration very copious; and in a very few days a change for the better was noticed. From which I must be allowed to believe that we may, without impropriety, attribute to the copaiba some agency in the cure.

In this case the urinary secretion was not much affected in respect to quantity, but a good deal so in smell and appearance.

Case III.—Mrs. Lay, aged seventy-three years, was attacked, a few weeks after the recovery of the preceding patient, with all the characteristic symptoms of inflammation of the mucous tissue of the lungs,—violent cough, sense of oppression, dull pain in the chest, hot skin, &c. The pulse being tense, venesection was had recourse to twice in the first twenty-four hours; after which a blister was applied over the chest.

In the course of the treatment, she was ordered small doses of antimonial preparations, and occasionally purged with Castor-oil. By means of these remedies, much benefit was obtained: her oppression and pain gradually disappeared, and her pulse, skin, and digestive organs, returned to their natural state. The cough continuing, however, and the expectoration being very copious, thick, opaque, and yellow, the remedies usually employed on such occasions were used several weeks, without effect: Balsam

Copaiba was therefore substituted, and administered in an emulsion, in doses of twenty-five drops three times a-day. Under the use of this medicine, the unpleasant symptoms gradually diminished, and at the end of less than a month had totally disappeared. Shortly after, however, Mrs. L. was attacked with all the symptoms of softening of the brain, which in a few days terminated fatally.

*Remarks*.—It is evident that in this case, notwithstanding its termination, the balsam copaiba was beneficial; since, under its use, the symptoms for which it was prescribed were entirely removed. The cephalic attack, from all appearances, was unconnected with the primary disease, and could not arise from the action of the copaiba.

Case IV.—Isabella S., about twenty-five years of age, of a lymphatic and bilious temperament, and subject for some time to frequent colds, was attacked, in December 1823, with the usual symptoms of catarrhal fever,—hot skin, frequent pulse, headache, attended with fullness and pain in the chest, and harassing cough. She was bled, puked with Ipecacuanha, ordered Antimonials in small and repeated doses, and demulcent drinks, and placed on a strict antiphlogistic diet. In a few days, the most urgent symptoms had in great measure subsided; but, the pain and cough continuing troublesome, a blister was ordered to the chest, Antimonials continued, and occasional doses of Castor-oil administered. This plan of treatment was persevered in for some time, when the fever diminished through the day, and only appeared towards night, attended with hot skin, quick pulse, and perspiration towards morning. The cough, however, still continued, was especially violent towards morning; and the expectoration was copious, thick, yellow, opaque, and at times of a greenish tinge.

Thinking that this opportunity was favourable for the administration of the Copaiba, it was prescribed in doses of twenty-five drops three times a-day, in a wine-glassful of milk, to be increased five drops each dose every third day, unless it produced irritation of the stomach, as indicated by nausea, pain, thirst, &c. Under the use of this remedy, occasional anodynes, and a milk diet, the cough and expectoration gradually disappeared, and in the course of a few months the girl recovered. She now enjoys good health.

Case V.—Sarah Morgan, aged nine years, of a lymphatic temperament, narrow chest, and who had laboured a few months before under an attack of bronchitis, was taken, in November 1824, with fever, loss of appetite, cough, very copious expectoration of white thick matter; not attended, however, with pain in the chest. She emaciated very rapidly; her skin became hot, her cheeks were flushed, especially at night; pulse much accelerated. In the course of ten or twelve days, she was puked several times with Ipecacuanha, purged with Castor-oil, ordered small doses of Antimonial Wine, and kept on low diet. Under this plan of treatment, she seemed to improve; the fever subsided, and no other symptoms appeared to remain, with the exception of her cough and copious expectoration. Mild tonics were now administered, and anodynes given every night to allay irritation; but under the use of these remedies the symptoms were aggravated: her pulse once more became accelerated, her skin hot, and the expectoration insensibly acquired a fetid smell and greenish tinge. It was now evident that the tonic plan, so far from being beneficial, had aggravated the irritation of the lungs. It was in consequence laid aside. Antimonial Wine was once more resorted to, and a blister applied to her chest. After a few days, the irritation of the system was calmed, but the cough and expectoration remained unabated.

Under these circumstances she was put under the use of Balsam Copaiba, in doses of six drops three times a-day; and anodynes were given at night to procure sleep. By persevering in the use of this remedy, and of a diet consisting of milk diluted with barley water, and a small portion of stale bread, Miss Morgan recovered perfectly in the course of six weeks. She had the benefit of the advice of Dr. Physick, who, at my suggestion, was requested to attend in consultation.

The following case I owe to the politeness of my friend Dr. Monges, of this city, a physician distinguished for his extensive experience and practical accuracy.

Case VI.—Mr. Roberts, aged twenty-two years, of a lymphatic temperament, and who had lost two brothers with phthisis pulmonalis, was attacked a few years ago with the usual symptoms of catarrh, which were soon so aggravated as to threaten him with consumption; the cough having become dry and harassing, and the fever gradually more intense and continual. For these Mr. R. was bled several times; cups, leeches, and blisters, were in succession applied to the chest, and occasional emetics prescribed. This plan was persevered in for three weeks, when, finding that Mr. R. was not benefited by it, and that his disease was assuming the same march as that which had proved fatal to his brothers, I resorted to mercury, in order, if possible, to arrest its progress. The gums soon became affected, and the salivation was kept up for six weeks. But from this powerful revulsive no benefit resulted. So far from this, indeed, the symptoms were much aggravated. The pulse was now 120 in a minute, the skin dry, cough frequent, expectoration very copious. Night sweats soon supervened; the lower extremities became œdematous, the debility extreme, and exercise occasioned difficulty of breathing. Under these circumstances Mr. Roberts was sent to the country, where he remained all summer, taking gum arabic and milk for nourishment, occasional purgatives, antimonial preparations, and squill.

When he returned from the country, he appeared worse: the fever had assumed the hectic character, the nocturnal sweats were more copious, his debility was greater than when he had left the city, and his features were so much altered that I did not recognise him. Milk was now discontinued, and nourishing food ordered; Balsam Copaiba in large doses was administered, and its cathartic effects prevented by opiates. This remedy had hardly been used two weeks, before the symptoms were in a great measure amended. The cough was better, the expectoration improved in appearance, the nocturnal fever less severe, and the sweats less copious. The balsam was continued three months, when a cure was effected.

At my suggestion, young Roberts abandoned the sedentary business of silversmith, which until that period he had followed, and adopted the active life of a farmer.

Three years have now elapsed since Mr. Roberts' first attack; and, although he has suffered from intermittent fever, and last spring from the influenza, his health is as good, and perhaps better, than before the severe attack in question.

*Remarks.*—It must be evident to every one that in this, as well as in the two preceding cases, the balsam copaiba was of the most decided benefit; as it put a stop to an irritation of the mucous membrane of the lungs, which, had it been allowed to continue much longer, might, owing to the predisposition of the patients arising from their temperaments, have occasioned the formation of tubercles, and ended in phthisis. For it is a well-established fact, that tubercles are, in almost all cases, the result of neglected inflammations of this membrane.

CASE VII.—J. E., about thirty-five years of age, of a strong constitution and bilious temperament, and by profession a mariner, was taken, whilst on the coast, with the usual symptoms of pulmonary inflammation. For the first four days, little or nothing was done to remove the disease; so that, when he came under care, (February 1826,) I entertained fears lest the inflammation had already progressed too far to be arrested. The pain was very considerable, the pulse strong and full, the oppression great, the cough almost continuous, and expectoration bloody. To remove these symptoms, the lancet was very freely and repeatedly had recourse to, warm cataplasms applied to the painful part, antimonials administered, &c. and the lowest possible diet enjoined. After a few days, the force of the attack appearing somewhat lessened by these means, a blister was applied to the chest, the antimonials continued, and occasional cathartics prescribed. This plan being persevered in for some weeks, the heat of the skin abated; but still the pulse was quick,. particularly at night, the pleuritic pain was not entirely removed, and the cough was troublesome, and attended with a copious expectoration of yellow or greenish matter. Swelling of the feet and face supervened, and, upon the whole, the case appeared a very unpromising one.

The gastro-enteric apparatus, however, appearing in good condition, as indicated by clean tongue, appetite, no thirst, and regular bowels, the Balsam of Copaiba was prescribed in the usual way; opiates were given to procure sleep; and, to obviate the extreme debility of the patient, milk and barley water, with a small portion of toasted bread, was allowed twice a-day. Tartar-emetic ointment was likewise ordered to be rubbed on the chest, to remove the pain remaining there; and produced a small crop of pustules.

Under this plan of treatment the patient continued about five weeks, and at the expiration of that time was considered so much improved in all respects, as to be allowed to use a more nourishing diet. The pain being still somewhat troublesome, external revulsives, and the balsam, were advised to be continued, and in a very short time succeeded in removing all traces of the disease. J. E. has since left this country in perfect health.

*Remarks.*—Although, in the treatment of this case, I did not avail myself of the aid of the stethoscope, I think it may be affirmed that I had to combat a conjoint inflammation of the pleura and of the mucous lining of the lungs; which, not having been arrested in the outset, had passed to a chronic state, and would, unless interfered with by proper means, have conducted the patient to the grave, with the usual symptoms of phthisis pulmonalis. Indeed, so unfavourable was my prognosis of the case, that I requested one of my most experienced medical friends to visit him once or twice with me, and his opinion corroborated mine as to the precarious situation of the patient; so true it is that physicians are very apt to be mistaken in their predictions respecting the issue of the most desperate case. Whether or not my opinion will be supported by that of my practical readers, I am disposed to attribute a considerable agency in this cure to the copaiba; as the benefit seemed to succeed closely its exhibition, and the case seemed closely allied, in all that relates to the mucous inflammation, to some others in which there could be no hesitation as regards the efficacy of this remedy. I would not, however, be thought to overlook the aid that was derived from the tartar-emetic ointment. To it, indeed, we must refer the removal of the pleuritic irritation.

Other cases might be adduced in confirmation of the advantage that may be derived from a judicious use of balsam copaiba in chronic inflammation of the mucous membrane of the lungs; but the fear of fatiguing the readers of this Journal, and extending beyond proper limits this already long article, compels me to restrict myself to these I have detailed. On some future occasion I shall resume the subject of this remedy, and offer some observations on its use in affections of the other portions of the same membrane.*

* North American Medical and Surgical Journal.

